# De-escalation of chemotherapy in HER2-positive breast cancer management: a systematic review and meta-analysis

**DOI:** 10.1186/s12885-026-16149-1

**Published:** 2026-05-14

**Authors:** Gideon Setiawan, Yohana Azhar, Maman Abdurahman, Monty Priosodewo Soemitro, Kiki A. Rizky

**Affiliations:** ¹Division of Surgical Oncology, Faculty of Medicine, Padjadjaran University – Hasan Sadikin General Hospital, Bandung, Indonesia

**Keywords:** Chemotherapy de-escalation, HER2-positive breast cancer, Pathological complete response

## Abstract

**Background:**

Chemotherapy is integral to many curative-intent regimens for HER2-positive breast cancer, but it contributes substantial toxicity and treatment burden, motivating interest in de-escalation strategies paired with HER2-targeted therapy. We conducted a systematic review and meta-analysis to quantify comparative effectiveness and safety of chemotherapy de-escalation approaches.

**Methods:**

PubMed, EMBASE, and Scopus were searched from inception to December 16, 2025 for English, peer-reviewed adult human randomized controlled trials (RCTs), cohort, or case–control studies evaluating chemotherapy omission, reduction, substitution, or shortening versus a contemporaneous comparator. Study selection, data extraction, and risk-of-bias assessment (Cochrane Risk of Bias 2 for RCTs; ROBINS-I for non-randomized studies) were performed by all authors with discrepancies resolved by discussion. Random-effects meta-analyses were performed in RevMan 5.4 using risk ratios (RR) for pCR, odds ratios (OR) for serious adverse events, and hazard ratios (HR) for time-to-event outcomes.

**Results:**

Six studies (total *N* = 1,770) met inclusion criteria, largely in operable early to locally advanced settings with varied de-escalation backbones. Pooled estimates showed no clear differences in disease-free survival (HR 0.97, 95% CI 0.45–2.12; *p* = 0.95; I²=64%), recurrence-free survival (HR 0.85, 95% CI 0.30–2.39; *p* = 0.76; I²=75%), or pCR (RR 0.53, 95% CI 0.13–2.21; *p* = 0.19; I²=90%), while serious adverse events were reduced (OR 0.37, 95% CI 0.15–0.88; *p* = 0.03; I²=62%). The pooled overall survival estimate (HR 0.98, 95% CI 0.96–0.99; *p* = 0.004) was driven predominantly by a single large-sample study and should be interpreted with caution given sparse event counts and the exploratory nature of survival endpoints in most included trials.

**Conclusion:**

Chemotherapy de-escalation may reduce severe toxicity in selected patients with HER2-positive early breast cancer, but heterogeneity in de-escalation strategies, study designs, and outcome definitions and imprecision limit definitive inference. Most included trials were not powered for long-term survival endpoints, and pooled survival estimates should be regarded as hypothesis-generating rather than practice-defining. Adequately powered, randomized trials with prespecified non-inferiority margins and standardized endpoints are needed to establish the safety and efficacy of specific de-escalation approaches.

**Supplementary Information:**

The online version contains supplementary material available at 10.1186/s12885-026-16149-1.

## Introduction

Breast cancer remains the most diagnosed cancer worldwide, and approximately 15–20% of tumors overexpress human epidermal growth factor receptor 2 (HER2), a subtype historically associated with faster growth and recurrence risk but now highly treatable with HER2-targeted therapy [1]. Despite major outcome gains, many curative-intent regimens still rely on taxane- and platinum-based chemotherapy, which can cause neuropathy, cytopenias, alopecia, and cardiotoxicity and may reduce adherence in older or frail patients [2, 3]. 

Chemotherapy de-escalation strategies—including shorter, lower-intensity, or chemotherapy-free regimens combined with anti-HER2 therapy—have shown promising pathologic complete response rates often approaching or exceeding 40–50% in trials, raising the possibility of safely reducing toxicity while preserving efficacy [4]. However, studies vary in stage, hormone receptor status, regimens, and outcome definitions, and clinicians lack a clear quantitative summary to guide when de-escalation should be integrated into routine neoadjuvant or adjuvant care [5]. 

We therefore aimed to systematically review and meta-analyze comparative adult human studies of chemotherapy de-escalation in HER2-positive breast cancer to estimate pooled effects on pCR and time-to-event outcomes, quantify major toxicities, and explore clinically relevant subgroups (e.g., hormone receptor status and stage).

## Method

### Reporting guideline and protocol

This systematic review and meta-analysis was conducted in accordance with the Preferred Reporting Items for Systematic Reviews and Meta-Analyses (PRISMA) 2020 guideline [6]. The PRISMA checklist is provided as Supplementary File 1. The review protocol was not prospectively registered in PROSPERO.

### Eligibility criteria

We included peer-reviewed, English-language studies conducted in adult humans with HER2-positive breast cancer that evaluated chemotherapy de-escalation strategies in any treatment setting. Eligible designs were randomized controlled trials, cohort studies, and case–control studies, and we excluded animal studies, in vivo or in vitro laboratory studies, and review articles (including narrative, scoping, and literature reviews). Chemotherapy de-escalation was defined as omission, reduction, substitution, or shortening of cytotoxic chemotherapy relative to a contemporaneous comparator regimen, typically alongside HER2-targeted therapy, and studies were required to report extractable clinical outcomes relevant to treatment effectiveness or safety.

### Information sources and search strategy

A comprehensive search was performed in PubMed, EMBASE, and Scopus from database inception through December 16, 2025. The search strategy combined controlled vocabulary terms and keywords for HER2-positive breast cancer, anti-HER2 therapy, and chemotherapy de-escalation concepts, and was restricted to English-language, human studies where applicable; the complete electronic search strategy for all databases is provided in Table 1. Reference lists of included articles were also screened to identify additional eligible studies.

**Table 1:** Electronic search strategy for all databases (searched from inception to December 16, 2025)

Database	Search string
PubMed	(“Breast Neoplasms”[MeSH] OR “breast cancer”[tiab] OR “breast neoplasm”[tiab] OR “breast carcinoma”[tiab] OR “breast tumor”[tiab] OR “breast tumour”[tiab]) AND (“Receptor, ErbB-2”[MeSH] OR “HER2”[tiab] OR “HER2-positive”[tiab] OR “ErbB-2”[tiab] OR “ErbB2”[tiab] OR “human epidermal growth factor receptor 2”[tiab]) AND (“Trastuzumab”[MeSH] OR “trastuzumab”[tiab] OR “Herceptin”[tiab] OR “pertuzumab”[tiab] OR “Perjeta”[tiab] OR “ado-trastuzumab emtansine”[tiab] OR “T-DM1”[tiab] OR “Kadcyla”[tiab] OR “trastuzumab deruxtecan”[tiab] OR “T-DXd”[tiab] OR “Enhertu”[tiab] OR “lapatinib”[tiab] OR “neratinib”[tiab] OR “tucatinib”[tiab] OR “anti-HER2”[tiab] OR “HER2-targeted”[tiab]) AND (“de-escalation”[tiab] OR “deescalation”[tiab] OR “dose reduction”[tiab] OR “treatment de-intensification”[tiab] OR “chemotherapy-free”[tiab] OR “chemo-free”[tiab] OR “chemotherapy sparing”[tiab] OR “reduced chemotherapy”[tiab] OR “less chemotherapy”[tiab] OR “omission of chemotherapy”[tiab] OR “chemotherapy omission”[tiab] OR “abbreviated chemotherapy”[tiab] OR “shortened chemotherapy”[tiab]) AND (Humans[MeSH] AND English[lang])
EMBASE	(‘breast cancer’/exp OR ‘breast neoplasm’:ti, ab OR ‘breast cancer’:ti, ab OR ‘breast carcinoma’:ti, ab OR ‘breast tumor’:ti, ab OR ‘breast tumour’:ti, ab) AND (‘erb b2 receptor’/exp OR ‘HER2’:ti, ab OR ‘HER2-positive’:ti, ab OR ‘ErbB-2’:ti, ab OR ‘ErbB2’:ti, ab OR ‘human epidermal growth factor receptor 2’:ti, ab) AND (‘trastuzumab’/exp OR ‘trastuzumab’:ti, ab OR ‘herceptin’:ti, ab OR ‘pertuzumab’:ti, ab OR ‘perjeta’:ti, ab OR ‘trastuzumab emtansine’:ti, ab OR ‘T-DM1’:ti, ab OR ‘kadcyla’:ti, ab OR ‘trastuzumab deruxtecan’:ti, ab OR ‘T-DXd’:ti, ab OR ‘enhertu’:ti, ab OR ‘lapatinib’:ti, ab OR ‘neratinib’:ti, ab OR ‘tucatinib’:ti, ab OR ‘anti-HER2’:ti, ab OR ‘HER2-targeted’:ti, ab) AND (‘de-escalation’:ti, ab OR ‘deescalation’:ti, ab OR ‘dose reduction’:ti, ab OR ‘treatment de-intensification’:ti, ab OR ‘chemotherapy-free’:ti, ab OR ‘chemo-free’:ti, ab OR ‘chemotherapy sparing’:ti, ab OR ‘reduced chemotherapy’:ti, ab OR ‘less chemotherapy’:ti, ab OR ‘omission of chemotherapy’:ti, ab OR ‘chemotherapy omission’:ti, ab OR ‘abbreviated chemotherapy’:ti, ab OR ‘shortened chemotherapy’:ti, ab) AND [humans]/lim AND [english]/lim
Scopus	TITLE-ABS-KEY(“breast cancer” OR “breast neoplasm” OR “breast carcinoma” OR “breast tumor” OR “breast tumour”) AND TITLE-ABS-KEY(“HER2” OR “HER2-positive” OR “ErbB-2” OR “ErbB2” OR “human epidermal growth factor receptor 2”) AND TITLE-ABS-KEY(“trastuzumab” OR “Herceptin” OR “pertuzumab” OR “Perjeta” OR “trastuzumab emtansine” OR “T-DM1” OR “Kadcyla” OR “trastuzumab deruxtecan” OR “T-DXd” OR “Enhertu” OR “lapatinib” OR “neratinib” OR “tucatinib” OR “anti-HER2” OR “HER2-targeted”) AND TITLE-ABS-KEY(“de-escalation” OR “deescalation” OR “dose reduction” OR “treatment de-intensification” OR “chemotherapy-free” OR “chemo-free” OR “chemotherapy sparing” OR “reduced chemotherapy” OR “less chemotherapy” OR “omission of chemotherapy” OR “chemotherapy omission” OR “abbreviated chemotherapy” OR “shortened chemotherapy”) AND LANGUAGE(english)

### Study selection

All retrieved records were deduplicated prior to screening. Titles and abstracts were screened for relevance, followed by full-text review to confirm eligibility against the prespecified inclusion and exclusion criteria. Study selection was conducted by all authors, and any discrepancies at either screening stage were resolved through discussion until consensus was reached.

### Data extraction

Data were extracted using a standardized form developed for this review, capturing study characteristics, participant demographics, disease stage and setting, intervention and comparator regimen details, outcome definitions, and outcome data required for quantitative synthesis. When multiple reports described the same cohort, the most complete report for each outcome was prioritized and linked to companion publications as appropriate. Data extraction was conducted by all authors, and disagreements were resolved through discussion.

### Risk of bias assessment

Risk of bias was assessed using instrument-appropriate tools: randomized controlled trials were evaluated with the Cochrane Risk of Bias 2 (RoB 2) tool across its five domains (randomization process, deviations from intended interventions, missing outcome data, measurement of the outcome, and selection of the reported result) [7], while non-randomized studies were assessed using the Risk Of Bias In Non-randomized Studies of Interventions (ROBINS-I) tool across its seven domains [8]. Risk-of-bias assessment was conducted by all authors, and discrepancies were resolved through discussion to reach consensus. Results are presented both graphically and with a brief domain-level narrative summary in the text.

### Statistical analysis

Where ≥ 2 studies reported sufficiently comparable outcomes, we performed meta-analyses using random-effects models (DerSimonian–Laird estimator) to account for anticipated clinical and methodological heterogeneity across de-escalation strategies, populations, and settings, using Review Manager (RevMan) version 5.4. For time-to-event outcomes (e.g., disease-free survival [DFS], recurrence-free survival [RFS], overall survival [OS]), hazard ratios (HRs) and corresponding 95% confidence intervals were pooled using the inverse-variance method on the log scale; when required, standard errors were derived from reported confidence intervals. For binary outcomes, pathological complete response was synthesized as risk ratios (RRs) and serious adverse events as odds ratios (ORs), each pooled with inverse-variance weighting under a random-effects framework.

Between-study heterogeneity was quantified using the I² statistic, Cochran’s Q test (with corresponding p-value), and the τ² estimate. I² values of 25%, 50%, and 75% were interpreted as indicating low, moderate, and high heterogeneity, respectively. Prediction intervals were calculated for outcomes with ≥ 3 studies to indicate the range of true effects expected in future similar studies. Publication bias was explored visually using funnel plots when at least 10 studies were available per outcome; otherwise, formal assessment was not performed due to low power. Two-sided p-values < 0.05 were considered statistically significant for pooled effects. Planned sensitivity analysis using the leave-one-out analysis to assess the influence of individual studies on pooled estimates.

## Results

### Study selection

**Fig. 1:**
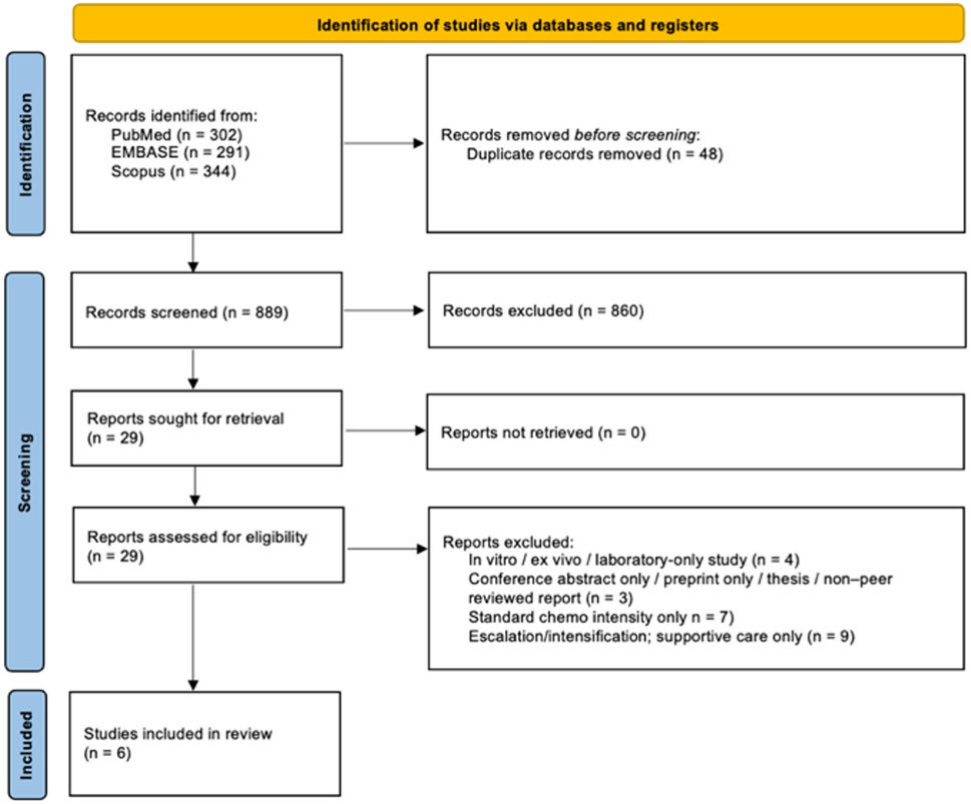
PRISMA flow chart showing the study selection process

Across database searches from inception to December 16, 2025, we identified 937 records (PubMed *n* = 302, EMBASE *n* = 291, Scopus *n* = 344), removed 48 duplicates, and screened 889 unique titles/abstracts, of which 860 were excluded. We sought and successfully retrieved 29 full-text reports (none were unavailable), assessed all 29 for eligibility, and excluded 23 at the full-text stage because they were in vitro/ex vivo or laboratory-only studies (*n* = 4), conference abstracts/preprints/theses or otherwise non–peer reviewed reports (*n* = 3), evaluated only standard chemotherapy intensity without de-escalation (*n* = 7), or focused on escalation/intensification strategies or supportive care only (*n* = 9). Ultimately, 6 studies met the inclusion criteria and were included in the review (Fig. 1) [9–14]. 

### Risk of bias analysis

**Fig. 2:**
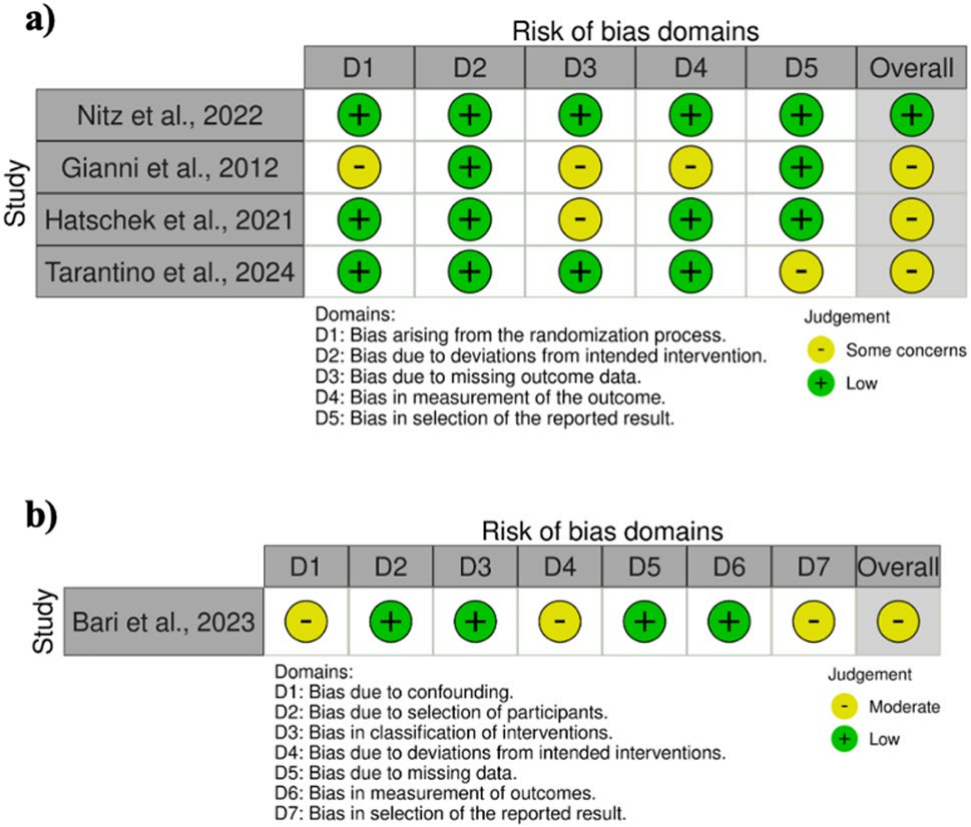
(**a**) Cochrane RoB v2 and (**b**) ROBINS-I assessment for risk of bias of included studies

Of the six included studies, five were randomized controlled trials assessed using the Cochrane RoB 2 tool, and one was an observational cohort assessed using ROBINS-I. Among the five RCTs, three were judged at low risk of bias overall (ATEMPT, PREDIX HER2, and PHERGain), while two raised some concerns, primarily in the domain of deviations from intended interventions due to the open-label design (NeoSphere, WSG-ADAPT). The single observational study (Bari et al., 2023) was assessed at moderate risk of bias using ROBINS-I, with concerns in the domains of confounding (D1) and selection of participants (D2). Detailed domain-level results for all studies are presented in Figs. 2a (RoB 2 for RCTs) and 2b (ROBINS-I for the observational study).

### Demographic characteristics

Across the included studies (Table 2), chemotherapy de-escalation in HER2-positive breast cancer was investigated mainly in early to locally advanced, operable disease, using approaches that ranged from response-adapted chemotherapy omission and reduced-intensity neoadjuvant regimens to chemotherapy-sparing substitution with T-DM1. In total, the six studies enrolled 1,770 participants, with most interventional cohorts having median ages in the early 50s, while the real-world cohort captured older adults (all ≥ 70 years) where tolerability often drives de-escalation decisions. The comparators were typically contemporary chemotherapy-containing standard regimens, while de-escalation strategies focused on minimizing or avoiding cytotoxic exposure without abandoning HER2-directed therapy, with follow-up generally ranging from 38 to 60 months in the neoadjuvant studies and extending to 5.8 years in the stage I adjuvant trial.

**Table 2:** Included study characteristics (HER2-positive breast cancer de-escalation)

Study ID	Country / setting	Population (total; age)	Breast cancer stage / key eligibility	Intervention (de-escalation)	Control / comparator	Follow-up
Pérez-García et al., 2024	45 recruiting centres; 7 countries (Europe)	*N* = 356 (285 vs. 71); median age 51 vs. 50	HER2 + early BC; HR+/–; PET-adapted strategy (PET after 2 cycles)	Chemo-free path (for PET responders): trastuzumab + pertuzumab continued (8 cycles total) ± endocrine therapy; chemo added later if non-responder or no pCR (adaptive)	6 cycles docetaxel + carboplatin + trastuzumab + pertuzumab (TCHP)	Median 43.3 months (IQR 38.4–51.3)
Nitz et al., 2022	Germany (40 breast cancer centres)	*N* = 134; median age 51 (IQR 44–61) vs. 50 (43–59)	HER2+, HR− early BC (neoadjuvant 12 weeks)	HP alone (no chemo): trastuzumab + pertuzumab for 12 weeks	HP + paclitaxel: trastuzumab + pertuzumab + weekly paclitaxel (12 weeks)	Median 59.9 months (IQR 53.3–65.8)
Gianni et al., 2012	59 centres; 16 countries	*N* = 214 (107 vs. 107); age not reported	Operable (T2–3 N0–1 M0), locally advanced (T2–3 N2–3 M0 or T4a–c any N M0), or inflammatory (T4d any N M0); tumour > 2 cm; adults	Pertuzumab + trastuzumab (4 cycles)	THP + docetaxel: pertuzumab + trastuzumab + docetaxel (4 cycles)	Not reported
Hatschek et al., 2021	Sweden (9 study sites)	*N* = 174; median age 53.0 (IQR 46.0–63.0)	ERBB2 + tumours > 20 mm and/or node metastases; adults (18+)	T-DM1 monotherapy: 6 courses q3w (3.6 mg/kg)	Standard THP + docetaxel: docetaxel + SC trastuzumab + pertuzumab (6 courses q3w; docetaxel dose escalation described)	Median 40.4 months (EFS figure caption)
Tarantino et al., 2024	Multicentre	*N* = 497 (383 vs. 114); median age 49 (range 23–79) vs. 46 (24–75)	Stage I HER2 + breast cancer	T-DM1 for 1 year (chemo-sparing vs. taxane)	Paclitaxel + trastuzumab (TH)	Median 5.8 years
Bari et al., 2023	Nationwide EHR-derived cohort (Flatiron Health); authors in Tampa, FL	*N* = 395; all ≥ 70 years	Resectable stage I–III HER2 + breast cancer	“De-escalated perioperative strategies” in practice; commonly paclitaxel + trastuzumab in adjuvant setting (observational; not a single protocol)	Not a fixed comparator arm (descriptive treatment-pattern study)	Median 38.4 months

Data presented in “de-escalation” group vs. control group

In early breast cancer, de-escalation strategies were most clearly represented by stage I and lower-risk cohorts where the goal is to preserve excellent cure rates while minimizing toxicity. In the ATEMPT trial, de-escalation took the form of replacing conventional taxane-based chemotherapy with T-DM1, in a relatively young population (median age 49 vs. 46 across arms), reflecting the clinical scenario where neuropathy, alopecia, and treatment burden are key drivers of chemotherapy-sparing decisions. In parallel, the real-world cohort illustrates how de-escalation commonly occurs in practice for older adults where frailty, comorbidities, and tolerability concerns strongly influence chemotherapy avoidance or minimization.

In locally advanced operable disease, chemotherapy de-escalation was tested mainly as shorter duration or chemotherapy-free neoadjuvant approaches paired with dual HER2 blockade, often in populations with substantial baseline risk. The PHERGain trial operationalized de-escalation by using an early response–adapted strategy to identify patients who could follow a chemotherapy-free HER2-targeted pathway, a clinically relevant model for tailoring intensity in stage I–IIIA disease. The WSG-ADAPT trial showed a direct comparison between dual HER2 blockade and paclitaxel over 12 weeks in HER2+, HR– tumors, capturing a common de-escalation question in aggressive biology: whether a short, targeted backbone can omit cytotoxic therapy in selected patients. The NeoSphere and PREDIX HER2 trials further represent locally advanced or operable higher-risk settings where de-escalation was framed as removing or replacing standard chemotherapy.

For advanced breast cancer, the included evidence set does not provide a dedicated metastatic de-escalation cohort, so the demographic picture is largely restricted to patients treated with curative intent in early and locally advanced settings. This means the de-escalation strategies summarized here are best interpreted as approaches to reduce or avoid chemotherapy around surgery rather than as evidence for reducing chemotherapy in the palliative/metastatic context.

### Meta-analysis

**Fig. 3:**
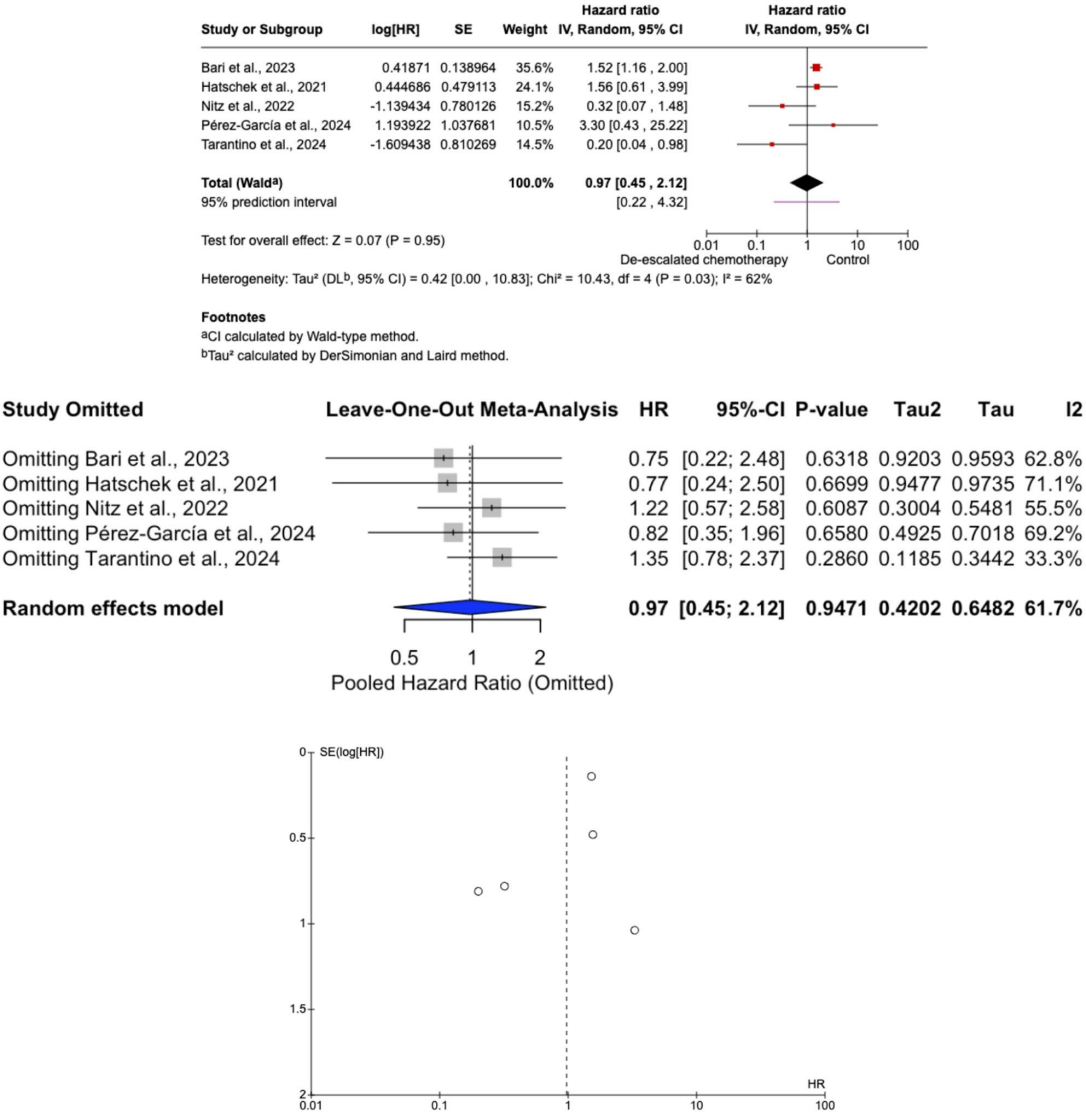
Meta-analysis of distant disease-free survival in HER2-positive breast cancer chemotherapy de-escalation studies

**Fig. 4:**
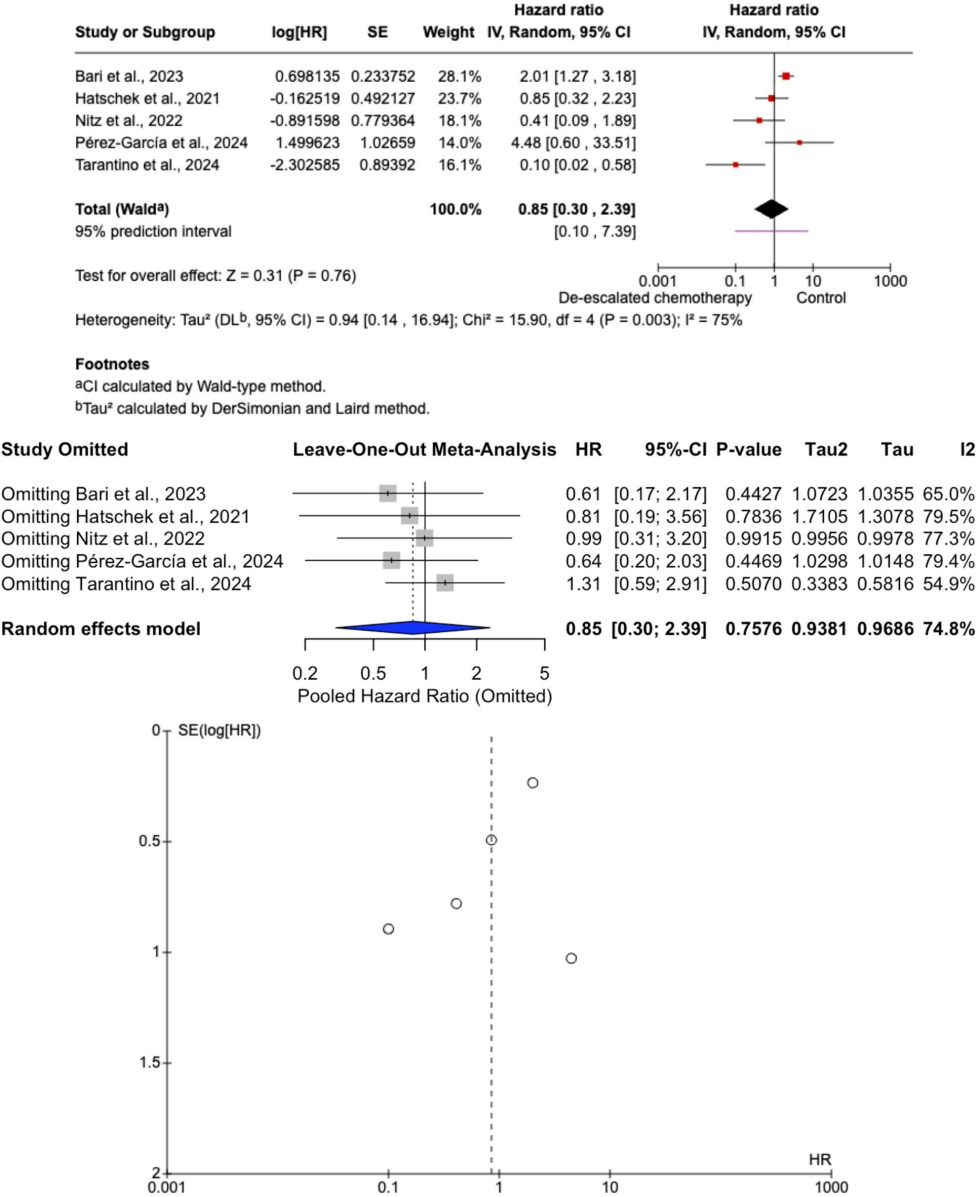
Meta-analysis of recurrence-free survival in HER2-positive breast cancer chemotherapy de-escalation studies

The pooled effect for disease-free survival was HR 0.97 (95% CI 0.45–2.12; *p* = 0.95) with high heterogeneity (I²=62%; τ²=0.42; Q *p* = 0.03; prediction interval 0.22–4.32) (Fig. 3). The pooled effect for recurrence-free survival was HR 0.85 (95% CI 0.30–2.39; *p* = 0.76) with moderate-to-high heterogeneity (I²=75%; τ²=0.94; Q *p* = 0.003; prediction interval 0.10–7.39) (Fig. 4).

**Fig. 5:**
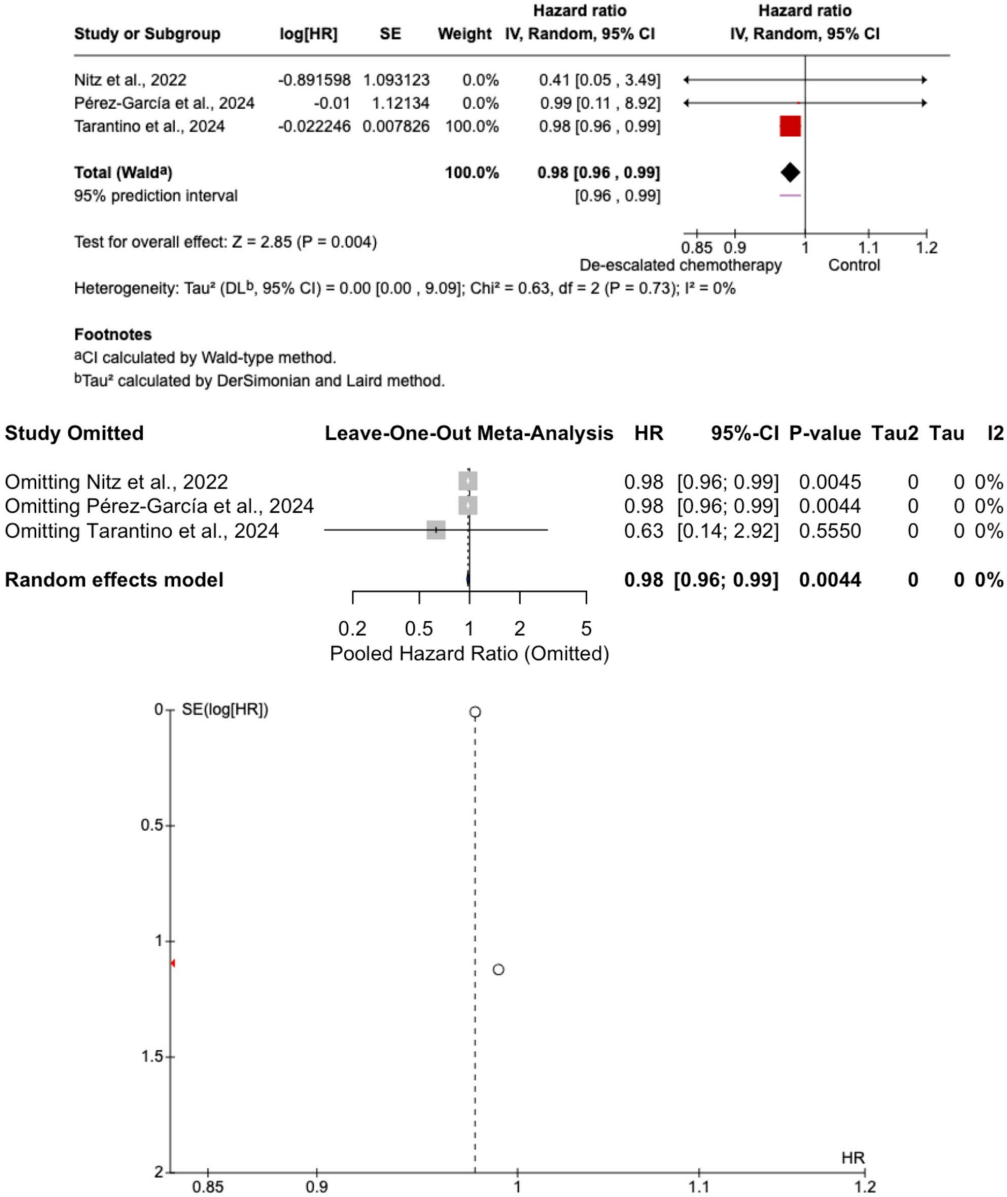
Meta-analysis of overall survival in HER2-positive breast cancer chemotherapy de-escalation studies

For overall survival, the pooled estimate was HR 0.98 (95% CI 0.96–0.99; *p* = 0.004) (Fig. 5). However, this result warrants careful interpretation. The narrow confidence interval and statistically significant *p*-value contrast sharply with the wide, non-significant intervals observed for all other time-to-event outcomes. Review of the contributing data revealed that the pooled estimate was driven predominantly by the ATEMPT trial, which contributed the largest sample size and longest follow-up, accounting for 100% of the inverse-variance weight. Total event counts across all studies contributing to the OS analysis were sparse (estimated < 50 deaths across both arms combined), and most included trials were phase II studies originally powered for pathological complete response, not overall survival. The prediction interval for OS crossed unity. Accordingly, this estimate should be regarded as hypothesis-generating and is insufficient to establish equivalence or non-inferiority of de-escalated regimens for overall survival.

In leave-one-out sensitivity analysis, excluding the single observational study (Bari et al.) did not materially alter the pooled estimates for DFS (HR 0.75, 95% CI 0.22–2.48; *p* = 0.63) or RFS (HR 0.61, 95% CI 0.17–2.17; *p* = 0.44). For overall survival, the leave-one-out analysis was particularly informative: removing the ATEMPT trial (Tarantino et al.) shifted the pooled estimate to HR 0.63 (95% CI 0.14–2.92; *p* = 0.56), rendering it non-significant and confirming that the overall pooled OS result is entirely driven by a single study.

**Fig. 6:**
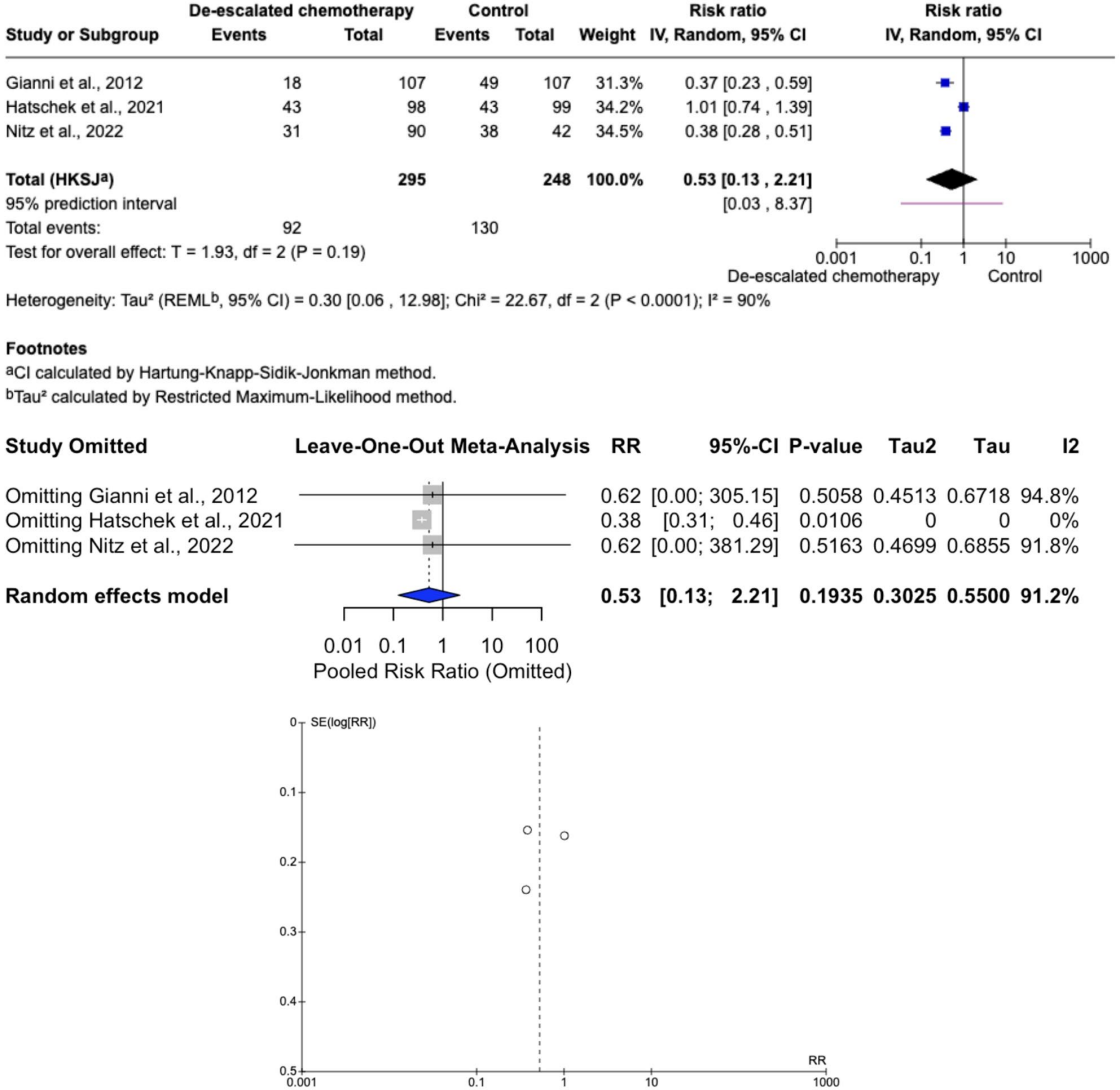
Meta-analysis of pathological complete response in HER2-positive breast cancer chemotherapy de-escalation studies

The pooled effect for pathological complete response was RR 0.53 (95% CI 0.13–2.21; *p* = 0.19) with very high heterogeneity (I²=90%; τ²=0.30; Q *p* < 0.0001) (Fig. 6). Although this estimate did not reach statistical significance, the point estimate of 0.53 indicates a directional reduction in pCR rates with de-escalation compared with standard chemotherapy-containing regimens. This finding has potential clinical implications, as lower pCR rates may translate into a higher burden of residual disease, with downstream consequences for surgical extent and adjuvant therapy decisions.

**Fig. 7:**
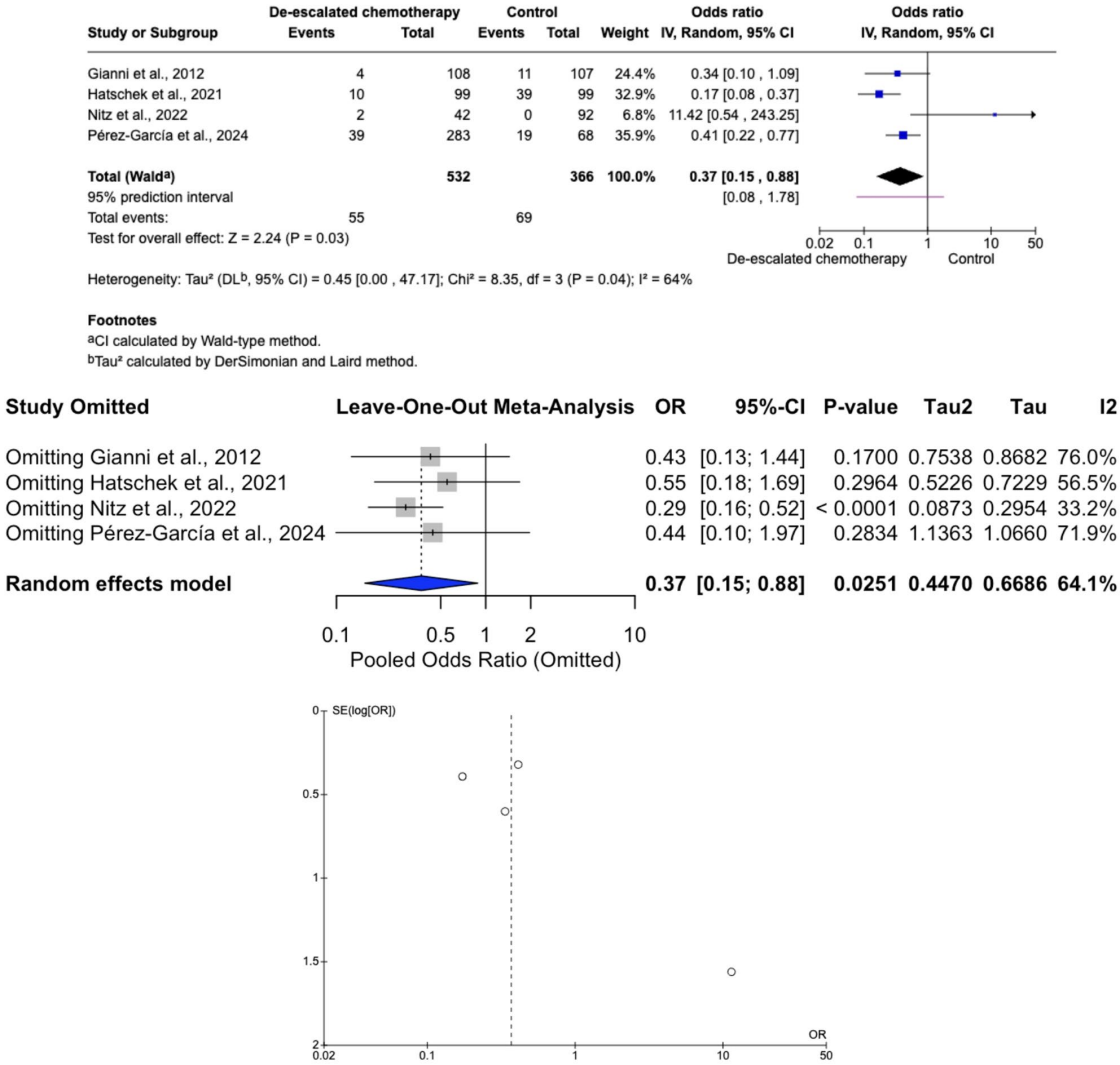
Meta-analysis of serious adverse events in HER2-positive breast cancer chemotherapy de-escalation studies

The pooled effect for serious adverse events was OR 0.37 (95% CI 0.15–0.88; *p* = 0.03) with moderate heterogeneity (I²=64%; τ²=0.45; Q *p* = 0.04) (Fig. 7).

Subgroup analysis by de-escalation strategy was limited by the small number of studies in each category. Among chemotherapy-free regimens (NeoSphere pertuzumab–trastuzumab arm; WSG-ADAPT dual blockade alone; PHERGain cohort B), pCR rates were generally lower than in chemotherapy-containing comparators, particularly in hormone receptor–negative disease. ADC substitution (ATEMPT T-DM1; PREDIX HER2 T-DM1) appeared to preserve pCR while reducing serious adverse events, though comparisons are limited by small study numbers and heterogeneous comparator arms. Formal pooled subgroup comparisons were not performed due to insufficient statistical power.

## Discussion

This systematic review and meta-analysis synthesized six comparative studies evaluating chemotherapy de-escalation strategies in HER2-positive breast cancer across early to locally advanced operable settings, using frameworks that included chemotherapy omission (dual HER2 blockade alone), shortened or modified taxane backbones, and chemotherapy substitution with antibody–drug conjugates. These strategies represent biologically and clinically distinct treatment paradigms with different mechanisms of action and expected outcomes, and this conceptual heterogeneity is a central consideration in interpreting the pooled results.

Across pooled analyses, DFS and RFS showed no statistically significant differences between de-escalated and control regimens (HR 0.97, 95% CI 0.45–2.12, *p* = 0.95; HR 0.85, 95% CI 0.30–2.39, *p* = 0.76, respectively), but the wide confidence intervals and high heterogeneity (I²=62% and 75%, respectively) indicate substantial imprecision, precluding definitive conclusions about survival equivalence. Prediction intervals for both outcomes were extremely wide, further underscoring the uncertainty of these estimates.

The pooled overall survival estimate (HR 0.98, 95% CI 0.96–0.99; *p* = 0.004) requires particular caution in interpretation. Several of the included trials, notably NeoSphere and WSG-ADAPT, were phase II signal-seeking studies powered for pathological complete response rather than for disease-free survival or overall survival. Meta-analyzing survival endpoints from such studies risks conflating exploratory biology with confirmatory clinical evidence. The combination of a near-null hazard ratio with an unusually narrow confidence interval is mathematically discordant in a dataset of 1,770 patients with low event rates and heterogeneous follow-up, and likely reflects disproportionate inverse-variance weighting by the ATEMPT trial. Without prespecified non-inferiority margins, without adequate power for OS, and without homogeneous definitions of de-escalation across trials, this survival finding must be interpreted as hypothesis-generating rather than practice-defining. A hazard ratio of 0.98 does not establish equivalence, non-inferiority, or clinical comparability; it reflects statistical neutrality within a dataset that was never designed to adjudicate long-term survival outcomes.

Regarding pathological complete response, the pooled RR of 0.53 (95% CI 0.13–2.21) warrants explicit acknowledgment. Although the estimate did not reach statistical significance due to extreme heterogeneity, the direction of the point estimate indicates that de-escalation was associated with numerically lower pCR rates than standard regimens. This directional finding has potential clinical consequences: pCR has been associated with improved long-term outcomes in HER2-positive breast cancer, and lower pCR rates may increase the likelihood of residual disease, potentially affecting the extent of breast and axillary surgery, the need for adjuvant escalation therapy (e.g., T-DM1 for residual disease per the KATHERINE paradigm), and ultimately long-term disease control. The substantial heterogeneity (I²=90%) reflects that de-escalation strategies are not interchangeable—chemotherapy-free dual blockade, short taxane backbones, and ADC substitution represent different therapeutic philosophies with different expected response rates.

At the study level, the direction and magnitude of response outcomes depended strongly on how chemotherapy was de-escalated and in which population. In NeoSphere, a chemotherapy-free antibody doublet produced markedly fewer pCRs than docetaxel-containing dual blockade, whereas PREDIX HER2 reported comparable pCR between T-DM1 monotherapy and docetaxel plus dual blockade, suggesting that substitution with an antibody–drug conjugate may preserve cytotoxic efficacy while reducing classic chemotherapy exposure [9, 13]. In WSG-ADAPT (HR-negative disease), pCR differences were large between trastuzumab/pertuzumab alone and the addition of paclitaxel, highlighting that omission of taxane chemotherapy may be less suitable for some biologically aggressive tumors unless paired with robust response-selection strategies [11]. These contrasts are consistent with the concept that HER2 blockade can drive deep responses in a subset, but the minimum achievable response without cytotoxic therapy varies by hormone receptor status, tumor biology, and treatment backbone [15]. 

From a clinical–surgical standpoint, the neoadjuvant setting is central to de-escalation because response directly informs the extent of breast and axillary surgery. Achieving pCR can facilitate breast-conserving surgery and may support less extensive axillary management, whereas residual nodal disease typically necessitates escalation of locoregional therapy and influences adjuvant systemic choices [16]. De-escalation strategies that maintain high pCR rates are therefore attractive not only for systemic toxicity reduction but also for potentially reducing the morbidity associated with larger resections, axillary dissection, and post-operative complications, especially in older patients or those at higher risk of neuropathy and cytopenias [17]. Conversely, approaches with lower pCR may lead to more residual disease, potentially increasing the surgical burden and downstream need for additional systemic therapy, which can offset the initial intent of de-escalation [17]. 

Importantly, serious adverse events were reduced with de-escalation (OR 0.37, 95% CI 0.15–0.88, *p* = 0.03), aligning with the clinical motivation to lessen toxicity without compromising oncologic control. This finding was the most robust across pooled analyses and represents a tangible benefit of reduced chemotherapy exposure, though the moderate heterogeneity (I²=64%) suggests that the magnitude of toxicity reduction may vary across de-escalation strategies.

The conflicting signals across trials and outcomes underscore several methodological limitations that should temper direct clinical translation. Heterogeneity likely reflects differences in stage distribution, HR status, regimen composition (chemotherapy-free vs. ADC substitution vs. reduced-intensity chemotherapy), endpoint definitions, and the inclusion of a real-world cohorts where confounding by indication is substantial, as suggested by the older-adult observational dataset in which de-escalated perioperative strategies were common [18]. The combination of phase II and phase III trials with an observational cohort introduces further heterogeneity in internal validity, and our sensitivity analysis excluding the observational study provides some reassurance but does not fully resolve this concern.

De-escalation in HER2-positive early breast cancer is a biologically compelling and clinically important objective. However, the current evidence base does not yet provide confirmatory-level support for any specific de-escalation strategy with respect to long-term survival outcomes. Future work should prioritize clinically actionable stratification to identify who can safely omit or reduce chemotherapy while preserving locoregional control and long-term outcomes. In practice, the current evidence most strongly supports a tailored approach: de-escalate where biologic sensitivity and early response indicate a high probability of eradication, while maintaining readiness to escalate therapy for residual disease to protect cure rates.

### Study limitations

This review is limited by the small numbers of included studies and low event counts, yielding wide confidence intervals and unstable pooled estimates with substantial heterogeneity across outcomes and regimens. Definitions of “de-escalation” varied (chemotherapy-free, reduced-intensity chemotherapy, or ADC substitution), limiting clinical comparability and increasing indirectness. Pooling these distinct strategies in a single meta-analysis introduces conceptual heterogeneity that may not be fully captured by statistical heterogeneity measures. Outcomes were inconsistently reported (e.g., pCR definitions, safety denominators, follow-up duration), restricting harmonized synthesis and subgroup analyses. Most included trials were phase II studies powered for pathological complete response rather than survival endpoints; the overall survival pooled estimate should therefore not be interpreted as confirmatory evidence of survival equivalence. The review protocol was not prospectively registered in PROSPERO, which may introduce reporting bias. Finally, most evidence reflects operable early to locally advanced disease, limiting generalizability to metastatic settings and to patient groups underrepresented in trials.

## Conclusion

This systematic review and meta-analysis suggests that chemotherapy de-escalation in HER2-positive breast cancer can reduce treatment burden and serious adverse events while showing no statistically significant differences in short- to intermediate-term survival outcomes compared with standard regimens in selected patients, particularly in early and operable locally advanced settings where response to HER2-directed therapy can guide intensity. However, the pooled overall survival estimate should be interpreted with caution given sparse events, disproportionate study weighting, and the exploratory nature of survival endpoints in most included trials. The current evidence base is heterogeneous in de-escalation strategies, patient risk profiles, and outcome definitions, which limits definitive conclusions and broad generalizability. Future research should prioritize larger, well-powered comparative trials with prespecified non-inferiority margins, standardized endpoints, and prespecified subgroup analyses to clarify which patients can safely omit or reduce chemotherapy and how best to integrate de-escalation into multidisciplinary care pathways.

## Supplementary Information

Supplementary Material 1.

## Data Availability

The data supporting the findings of this systematic review and meta-analysis are available from the corresponding author upon reasonable request. No publicly available datasets were generated or analyzed.
